# The Role of Mitochondrial Dynamin in Stroke

**DOI:** 10.1155/2022/2504798

**Published:** 2022-05-06

**Authors:** Chenchen Li, Chunli Chen, Haiyun Qin, Chuncao Ao, Jinlun Chen, Jieqiong Tan, Liuwang Zeng

**Affiliations:** ^1^Department of Neurology, Second Xiangya Hospital, Central South University, Changsha, Hunan 410011, China; ^2^Center for Medical Genetics, School of Life Sciences, Central South University, Changsha, Hunan 410078, China; ^3^Hunan Key Laboratory of Medical Genetics, Central South University, Changsha, Hunan 410078, China; ^4^Hunan Key Laboratory of Animal Model for Human Diseases, Central South University, Changsha, Hunan 410078, China

## Abstract

Stroke is one of the leading causes of death and disability in the world. However, the pathophysiological process of stroke is still not fully clarified. Mitochondria play an important role in promoting nerve survival and are an important drug target for the treatment of stroke. Mitochondrial dysfunction is one of the hallmarks of stroke. Mitochondria are in a state of continuous fission and fusion, which are termed as mitochondrial dynamics. Mitochondrial dynamics are very important for maintaining various functions of mitochondria. In this review, we will introduce the structure and functions of mitochondrial fission and fusion related proteins and discuss their role in the pathophysiologic process of stroke. A better understanding of mitochondrial dynamin in stroke will pave way for the development of new therapeutic options.

## 1. Introduction

Stroke is divided into ischemic stroke and hemorrhagic stroke [1]. 85% of strokes are ischemic, while hemorrhagic stroke accounts for 15% of all strokes [2, 3]. Ischemic stroke is caused by cerebral artery occlusion [2]. Currently, intravascular thrombectomy and tissue plasminogen activator (tPA) are the only recognized methods for the treatment of ischemic stroke [4, 5]. However, tPA therapy is still limited by the narrow treatment window [6]. Therefore, it is necessary to find a new treatment strategy for ischemic stroke [7, 8]. Ischemic stroke involves a series of pathological processes, including ischemia, hypoxia, destruction of calcium homeostasis, free radical production, edema, and excitotoxicity [9–11]. Various subcellular organelles play roles in the pathogenesis of ischemic stroke. The dysregulation of calcium homeostasis can cause unfolded and misfolded proteins increased, resulting in endoplasmic reticulum (ER) stress [12, 13]. Golgi apparatus had obvious morphological changes in cerebral ischemia model [14]. Lysosomes are also involved in ischemic neuronal cell death, characterized by increased lysosomal enzyme activity [15]. In addition, mitochondria dysfunction plays a key role in the pathological course of ischemic stroke [16].

Intracerebral hemorrhage (ICH) refers to the primary, spontaneous, and nontraumatic hemorrhage occurring in the brain parenchyma [17]. At present, there is no effective treatment for ICH. Hematoma evacuation is used in some patients, but the effect is not satisfactory [18]. In addition to the primary injury caused by hematoma, secondary injury plays a vital role in the further injury of ICH [19]. The mechanism of secondary brain injury caused by ICH is very complex, which mainly involves the following aspects: inflammation, neuronal apoptosis and necrosis, oxidative stress, the generation of reactive oxygen species (ROS), mitochondrial function impaired and so on [20, 21]. Mitochondrial dysfunction may play an important role in pathophysiological process of intracerebral hemorrhage.

Mitochondria are extremely dynamic organelles, which involves constantly dividing and elongating [22]. Mitochondrial fission and fusion may be related to neuronal cell death [23]. Mitochondrial dynamics can not only maintain mitochondrial DNA integrity, oxidative respiratory chain and intracellular calcium signal transduction but is essential in the process of apoptosis and neuronal death [24, 25]. Mitochondrial dysfunction is one of the markers of ischemia/reperfusion injury and subsequent neuronal death. Therefore, targeting the mitochondria is one of the strategies for the treatment of stroke [26]. In this review, we will discuss the role of mitochondria in stroke and focus on the effect of mitochondrial dynamin on the damage and recovery of neurons in stroke.

## 2. Mitochondrial Dysfunction in Stroke

### 2.1. Mitochondrial Dysfunction in Ischemic Stroke

Cerebral artery occlusion is the initial event of ischemic stroke, accompanied by severe oxygen and glucose deprivation (OGD) and a series of pathological processes, which eventually lead to irreversible cerebral injury and neuronal death [10, 27]. The initial loss of blood supply disrupts the neuronal functions and makes it unable to maintain normal transmembrane ion gradient, resulting in neuronal depolarization [9]. In the process of depolarization, neurons release glutamate, which leads to further depolarization of adjacent neurons [28]. Glutamate receptors, including N-methyl-D-aspartate-receptor (NMDAR), *α*-amino-3-hydroxy-5-methyl-4-isox-azolepropionic acid receptor (AMPAR), and Kainic acid receptor (KAR), are overactivated, resulting in the influx and accumulation of Ca^2+^ into cells [29–31]. A large amount of Ca^2+^ influx leads to excessive production of ROS and mitochondrial dysfunction, including imbalance of mitochondrial fission and fusion, mitochondrial-induced apoptosis, and mitophagy [27]. Simultaneously, accumulation of intracellular Ca^2+^ results in the cleavage of Bcl-2 interaction domain into truncated bid [32], which interacts with proapoptotic proteins on the membrane of mitochondrial and thus causing the mitochondrial permeability transition pores (MPTP) opening [33]. MPTP opening leads to a decrease in mitochondrial membrane potential (*Δψ*m), allows the release of cytochrome c or apoptosis-inducing factor (AIF)[34], and induces Ca^2+^ deregulation [35], thus leading to the activation of effector caspase and eventually the death of neurons [36, 37]. The imbalance of mitochondrial fission and fusion plays a vital role in neuronal death.

In addition to the adverse effects on neurons, ischemia-induced injury seriously affects glial cells, including oligodendrocytes, microglia, and astrocytes [38]. Neurons interact structurally with glial cells in the brain. Various types of glial cells play a vital role in regulating neuronal physiology and functions. Oligodendrocytes, myelin-forming cells in the central nervous system, are particularly sensitive to insults including hypoxia, reactive oxygen and nitrogen species and excitotoxicity [39, 40], which impair the functional activity of mitochondrial respiratory chain [41]. Microglia, macrophages in the brain, are critical for regulating neuroinflammation. Mitochondrial homeostasis plays an essential role in maintaining the function of microglia, and its imbalance is involved in the pathological process of ischemic stroke [42]. Several studies have demonstrated that excessive mitochondrial fission was observed in microglia during neuroinflammatory response [43, 44]. Astrocytes, the most abundant subtype of glial cells, are structural elements of gap junction and blood-brain barrier (BBB) and maintain multifaceted brain functions through communication with neurons [45]. Astrocytes protect neurons from hypoxia and excitotoxic injury [46, 47], while inhibiting astrocyte mitochondria function makes neurons susceptible to cell death [48]. Therefore, mitochondrial dysfunction plays an extremely important role in the pathogenesis of ischemic stroke.

### 2.2. Mitochondrial Dysfunction in Intracerebral Hemorrhage

The pathological process of ICH includes primary brain injury and secondary brain injury [21]. Secondary brain injury including elevated intracranial pressure or hydrocephalus may be the main factors leading to poor prognosis of ICH [49]. Mitochondrial dysfunction may impair the generation of adenosine triphosphate (ATP) and then lead to the failure of cellular pumps, resulting in cytotoxic edema and neuronal death [50, 51]. Mitochondrial dysfunction leads to the destruction of ROS homeostasis, which further results in mitochondrial damage, and the vicious cycles continues [52]. Excessive ROS will break the balance between ROS and antioxidant system, which leads to DNA damage and lipid peroxidation, initiates apoptosis, and impairs the BBB, resulting in brain damage [53, 54]. It is reported that mitochondrial dynamic improvement can prevent neurological deficits after subarachnoid hemorrhage (SAH) [55, 56]. Therefore, protecting mitochondrial function may be a potential target for the treatment of hemorrhagic stroke injury [57].

## 3. The Role of Mitochondrial Dynamin

Mitochondria are dynamic organelles where homeostasis can be regulated by complicated processes, including fission and fusion, biogenesis, and elimination [58, 59]. Mitochondrial dynamics are regulated by many proteins (Table 1) [60]. Dynamin-related protein 1 (DRP1) and mitochondrial fission protein 1 (FIS1) are key regulators of fission, while mitofusin-1/2 (MFN1/MFN2) and optic atrophy 1 (OPA1) are key regulators of fusion [61–63]. Fusion of mitochondrial outer membranes is mediated by MFN1 and MFN2, while fusion of mitochondrial inner membrane is mediated by OPA1 [64]. Fission is responsible for isolating dysfunctional mitochondria that contain damaged proteins, unstable membranes, and damaged or mutated mitochondrial DNA [60, 65–68]. Fusion facilitates the complementation between neighboring mitochondria, enabling survival of damaged mitochondria [69]. Mitochondrial dynamics emerges as a crucial process in regulating cell survival and death. Mitochondrial fission precedes neuronal death after cerebral ischemic stroke [70–72]. In the review, we specifically focused on the role of mitochondrial dynamic protein in regulating the stability of mitochondrial internal environment in stroke.

## 4. Mitochondrial Fission Proteins and Stroke

### 4.1. DRP1

DRP1 is a highly conserved dynamin which is essential for mitochondrial biogenesis and maintenance of normal mitochondrial homeostasis and integrity [69]. DRP1 is recruited by FIS1, mitochondrial fission factor (MFF), and N-terminally anchored mitochondrial dynamic proteins of 49 and 51 kDa (MID49/51). Under normal conditions, DRP1 is translocated from the cytoplasmic pool to the outer membrane of mitochondria, where DRP1 is assembled into constrictive ring-like multimeric structures, and ultimately drives mitochondrial fission through a GTP-dependent mechanism [73–75]. However, different stress conditions, including oxidative stress and hypoxia, lead to up regulation of DRP1 expression, and increased translocation of cytosolic DRP1 to mitochondria, causing imbalance between mitochondrial fission and fusion, which results in mitochondrial disintegration and concomitant apoptosis [76, 77]. In addition, excessive fission is associated with a variety of neurodegenerative diseases, especially stroke [94].

Mitochondrial fission is the initial event of apoptotic cell death after ischemic stroke [70–72]. DRP1 mediated mitochondrial oxidative damage is involved in the pathological process of acute nerve injury induced by ICH [95]. Aberrant regulation of mitochondrial dynamics following stroke shifts the balance of mitochondria fission and fusion towards fission, causing neuronal injury [42, 56]. DRP1, as the main mediator of mitochondrial fission, plays an important role in stroke. FIS1, MFF, and the proapoptotic Bcl-2 family protein Bax [96, 97] act directly or indirectly as DRP1 receptors to promote mitochondrial fission. DRP1 translocation and associated mitochondrial fission are key features of mitochondrial membrane potential (MMP) loss and neuronal cell death [98]. Several studies have reported that excessive mitochondrial fission in neurons is related to ischemic stroke, and inhibiting mitochondrial fission can reduce mitochondrial autophagy and neuronal apoptosis induced by ischemia/reperfusion injury [99].

#### 4.1.1. Expression of DRP1 in Stroke

Abundant experimental evidence has demonstrated that the activation of DRP1 and mitochondrial fission contributes to cerebral ischemic injury. He et al. [100] have observed an increased level of DRP1 in mice subjected to cerebral ischemia and reperfusion injury. After the knockdown of DRP1, oxidative stress, mitochondrial ROS production, and the infarct volume are decreased, which contribute to the survival of neurons in cerebral ischemia. Antioxidants such as vitamin E or MitoQ can reduce DRP1 expression and mitochondrial fragments [101, 102]. DRP1 inhibitors can prevent OGD-induced death and glutamate excitotoxicity in primary cultured neurons, and reduce the infarct size in cerebral ischemia model in vivo [71]. Zhao et al. [72] showed that in middle cerebral artery occlusion (MCAO) mice, inhibition of DRP1 not only attenuates OGD induced cell death but also significantly reduces infarct size and neuronal death by inhibiting Bax insertion into the outer mitochondrial membrane and oligomerization and subsequent release of cytochrome *c*. Inhibition of DRP1 can prevent the morphological changes of mitochondria and subsequent cell death [71]. Inhibiting DRP1 by mdivi1 reduced ischemic injury, it paradoxically increased damage during reperfusion [103, 104]. Therefore, inhibition of DRP1 may be a potential therapeutic strategy for neurological diseases including ischemic stroke.

The DRP1 expression has been studied in hypoxia models of hyperglycemia, but results are controversial. Zuo et al. [105] revealed that hyperglycemia inhibit the level of DRP1 in cytoplasm and mitochondria, which caused the accumulation of damaged mitochondria and subsequent injury. Hyperglycemia altered self-adaptation by shifting permanent MCAO (pMCAO) induced morphological changes to fusion. And upregulation of DRP1 significantly reduced the infarct volume, decreased the release of cytochrome *c*, inhibit the activation of caspase-3, and reduced the production of ROS in hyperglycemia induced cerebral ischemia injury. It is suggested that DRP1 has protective effect on cerebral ischemia injury induced by hyperglycemia. Transient focal cerebral ischemia/reperfusion can upregulate DRP1, implicating that the dynamic imbalance of mitochondrial fission was involved in the mediating ischemic neuron injury. Paradoxically, Liu et al. [106] have demonstrated that diabetic hyperglycemia further aggravated the upregulation of DRP1 after MCAO, which indicate that diabetes further inclined mitochondrial dynamic to fission. The difference between these studies could be explained by the different ischemia times in the models. Zuo et al. used a permanent cerebral ischemia model and focused on the relationship between hyperglycemia after ischemia and mitochondrial homeostasis in the early stage of ischemia (within 6 h clinical treatment window of thrombolysis). However, Liu et al. emphasize on exploring the effect of hyperglycemia on cerebral ischemia/reperfusion injury. Notably, hyperglycemia is a well-known harmful factor in ischemic injury. Clinically, the intervention of blood glucose is expected to become a therapeutic target for cerebral ischemic injury aggravated by hyperglycemia.

There are some studies about the DRP1 expression level after ICH injury. The critical pathogenic factor of secondary injury after ICH, acrolein, causes increased mitochondrial DRP1 translocation and further excessive mitochondrial fission, while the acrolein scavenger significantly suppressed the expression of DRP1 and alleviated mitochondrial morphological damage after ICH, leading to significant amelioration of brain edema, neural apoptosis, and neurological deficits [95]. Therefore, excessive DRP1 activity may be an important pathogenic factor of ICH injury.

#### 4.1.2. DRP1 Modification in Stroke

**Figure 1:**
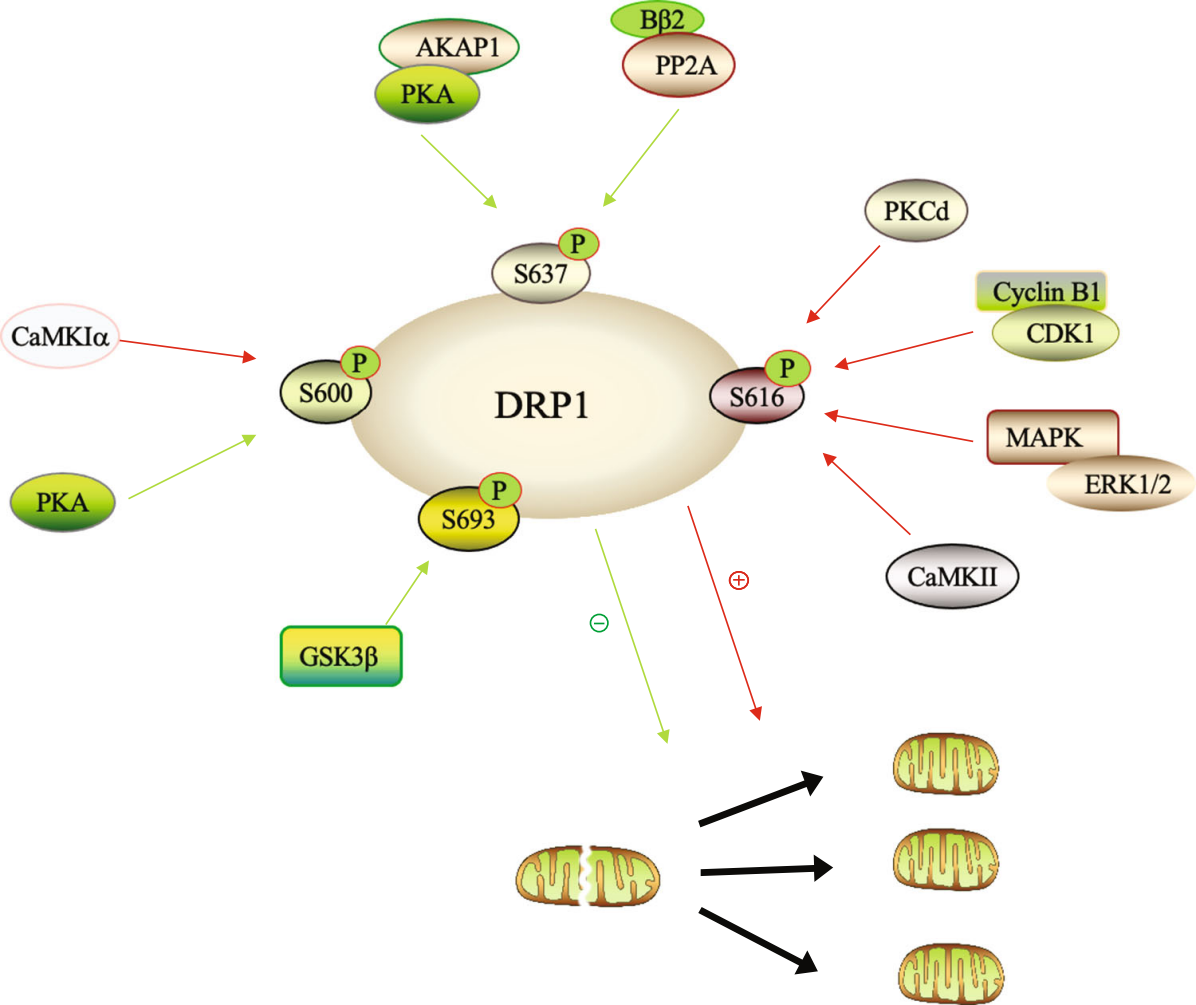
The phosphorylation modification of DRP1 in stroke. DRP1 has four major phosphorylation sites, including serine residues 600, 616, 637, and 693. DRP1 phosphorylation at S616 facilitates mitochondrial fission, whereas DRP1 S637 and S693 phosphorylation inhibits it. DRP1 Ser600 can be phosphorylated by PKA to reduce the activity of DRP1 GTPase; however, phosphorylation of the same serine residues by CaMKI*α* facilitate mitochondrial fission. PKA: protein kinase A; CaMKI*α*: Ca2 + -calmodulin dependent protein kinase I*α*; PKCd: protein kinase Cd; CDK1: cyclin-dependent kinase 1; MAPK/ERK1/2: mitogen-activated protein kinase/extracellular signal-regulated kinase 1/2; CaMKII: calmodulin-dependent protein kinase II; AKAP1: A-kinase anchoring protein 1; PP2A: protein phosphatase 2A; GSK3*β*: glycogen synthase kinase-3 beta.

DRP1 is subjected to a variety of posttranscriptional modifications, including S-nitroylation, SUMOylation, ubiquitination, and phosphorylation, which can activate or inhibit fission activity, and phosphorylation is the main modification [107, 108]. At present, four major phosphorylation sites of DRP1 have been found, which are located at serine residues 600, 616, 637, and 693 of DRP1 [78, 79, 109–111]. DRP1 phosphorylation at S616 facilitates mitochondrial fission, whereas DRP1 S637 and S693 phosphorylation inhibits it [110, 112] (Figure 1).

DRP1 Ser600 can be phosphorylated by protein kinase A (PKA), which can reduce the activity of DRP1 guanosine triphosphatase (GTPase) and decrease recruitment of DRP1 to mitochondria [78, 113]. However, phosphorylation of the same serine residues by Ca^2+^-calmodulin-dependent protein kinase I*α* (CaMKI*α*) resulted in significant increase in mitochondrial fission [109]. In addition, the phosphorylation of Ser616 can activate DRP1 and promote mitochondrial fission [114–117]. DRP1 at S616 can be phosphorylated by multiple kinases, including protein kinase Cd(PKCd), mitogen-activated proteinkinase (MAPK)/extracellular signal-regulated kinase (ERK)1/2, cyclin-dependent kinase 1 (CDK1)/cyclin B1, and calmodulin-dependent protein kinase II (CaMKII) [114, 115, 118].

The phosphate regulation of DRP1-S637 plays an essential role in determining ischemic sensitivity[119]. S637 is the inhibitory phosphorylation site of DRP1. Dephosphorylation of S637 can activate DRP1, promoting mitochondrial fission. As the first identified A-kinase anchoring protein (AKAP), AKAP1 can bind to the regulatory subunits of protein kinase A (PKA), which enables targeting of PKA holoenzyme to mitochondria [120, 121], and orchestrate coordinating the translational and posttranslational regulation of mitochondrial localization proteins. DRP1 is one of the key targets of AKAP1/PKA signaling complex. PKA mediates the phosphorylation of DRP1 at Serine 637 (S637 in human subtype 1 and S656 in rat subtype 1) and inhibits its fission activity [78, 113]. The outer mitochondrial AKAP1/PKA complex inhibits DRP1-dependent mitochondrial fission and protects neurons from ischemic stroke. AKAP1 is rapidly degraded following hypoxia [122–124], which impaired the inhibitory phosphorylation site S637 of DRP1 and enhanced mitochondrial localization of fission enzyme, promoting mitochondrial fission and the localization of DRP1 to mitochondria. However, deletion of AKAP1 did not alter overall level of DRP1.The neuron specific DRP1 activator, B*β*2, is a mitochondrial-localized protein phosphatase 2A (PP2A) regulatory subunit. Deletion of B*β*2 and maintenance of DRP1 Ser637 phosphorylation can improve mitochondrial respiratory capacity, maintain Ca^2+^ homeostasis, reduce superoxide production under ischemic and excitotoxic conditions, and rescue excessive stroke injury [94]. The phosphorylation regulation of DRP1-S693,which also inhibiting mitochondrial fission, plays an important role in ischemic stroke, and is related to the control of mitophagy induction [125]. As Chou et al. [110] revealed that DRP1 is phosphorylated by glycogen synthase kinase-3 beta (GSK3*β*) at Ser693, resulting in rapid elongation of mitochondria through attenuation of GTPase activity and downregulation of cytochrome c release, caspases-3 and -7, which plays an anti-apoptotic role and alleviates ischemic injury.

In the pathological process of ICH injury, acrolein regulates the translocation of DRP1, which is partially dependent on ERK and AMP-activated protein kinase (AMPK) pathways, targeting DRP1 phosphorylation at Ser616 and Ser637, respectively [95]. Excessive DRP1-mediated fission will damage mitochondrial structure, resulting in impaired respiratory chain function, ATP deficiency, increased generation of mitochondrial ROS, and activation of apoptosis pathways [126, 127]. DRP1 inhibitor, mdivi-1, can ameliorate oxidative stress injury and neuronal apoptosis caused by subarachnoid hemorrhage [55, 128]. Several studies have shown that DRP1-mediated mitochondrial fission is usually accompanied by an intense inflammatory response [129]. The recruitment of DRP1 to mitochondria after ICH impairs mitochondrial morphology and astrocyte function, which can be rescued by adiponectin peptide, a crucial upstream regulator of DRP1, in vivo and in vitro [130]. Notably, when selective inhibition of AMPK pathway, a signal pathway regulating DRP1 phosphorylation at Ser637, it can be observed that adiponectin peptide-mediated protection against astrocytic inflammation and DRP1-mediated mitochondrial fission is inhibited [130]. The overexpression of tyrosine kinase Fyn participates in neuroinflammation, oxidative stress, and apoptosis, amplifies the up regulation of p-ser616 DRP1, and reduces the antiapoptotic protein induced by ICH, which can be reversed by mdivi-1 [131]. Therefore, phosphorylation at different sites regulated by kinases affects mitochondrial recruitment of DRP1, indicating different functional and morphological effects, and the regulation of DRP1 phosphorylation at different sites is a potential therapeutic target against the stroke injury.

#### 4.1.3. DRP1 and Neuroinflammation

Neuroinflammation is one of the main causes of ischemic stroke, and reducing neuroinflammation can inhibit stroke-related injuries [132]. Neuroinflammation is accompanied by abnormal activation of JAK2/STAT3 pathway [133, 134]. DRP1 S616 appears to be associated with neuroinflammation. Anti-inflammatory drug atractylenolide III regulates neuroinflammation and improves ischemia/reperfusion injury by inhibiting JAK2/STAT3 pathway and reducing DRP1 translocation and phosphorylation at Ser616 to restrain mitochondrial fission [42]. Phosphorylated DRP1 can translocate to the outer membrane of mitochondria, where it interacts with FIS1 to induce mitochondrial fission, then producing ROS and activating nucleotide-binding domain- (NOD-) like receptor protein 3 (NLRP3) inflammasome [135]. Moreover, ketogenic diets (KD) may inhibit the mitochondrial translocation of DRP1 to suppress NLRP3 inflammasome activation, which may suppress ER stress and protect mitochondrial integrity, thus exerting neuroprotective effects in ischemic stroke [135].

#### 4.1.4. DRP1 in Apoptosis and Autophagy

Multiple studies have reported that DRP1 translocation and increased mitochondrial fission are associated with apoptosis [96, 136, 137]. Inhibiting apoptosis can reduce ischemic injury [138]. Mitochondria can be degraded by mitophagy (selective) and bulk autophagy (nonselective), both of which may be caused by nutritional deficiency, starvation, or rapamycin treatment [139–141]. Autophagy is a process of degrading misfolded proteins and damaged organelles [142, 143]. Autophagy occurs in mitochondria and selectively removes damaged mitochondria, which is called mitophagy. Once disrupted, the damaged mitochondria would accumulate in the cellular, resulting in the release of a variety of mitochondrial proteins from the mitochondrial matrix to the cytoplasm, which leads to neuronal apoptosis. In some cases, mitophagy may be induced independently of bulk autophagy. In hyperglycemic animals, bulk autophagy increased and mitophagy decreased. Hyperglycemia elevated the autophagy level and maintained them at a high level after pMCAO. Since mitophagy can be induced by mitochondrial fission, inhibition of DRP1 can prevent the clearance of damaged mitochondria by mitophagy following pMCAO[104]. The harmful effect of blocking mitochondrial fission is due to the inhibition of mitophagy, which prevented the removal of damaged or defective mitochondria.

Over activation of DRP1 activity seems to be a crucial pathogenic factor of stroke injury. However, fragmented mitochondria are more likely to be eliminated by autophagosomes and then protecting cells from death [144]. In view of different disease models in different pathological processes, it is unclear whether DRP1-mediated mitochondrial fragmentation contributes to autophagy and neural survival after ICH. It is worth noting that autophagy may prevent the induction of apoptosis; however, excessive autophagy contributes to aggravate neural injury and induce apoptosis [145].

### 4.2. FIS1

FIS1 is the only DRP1 recruitment factor and can bind to human DRP1 in vitro [80]. When FIS1 is overexpressed, it can promote fission and participate in many fission dependent processes, including apoptosis and autophagy [81–83]. The deletion of FIS1 resulted in mitochondrial hyperfusion, mitochondrial dysfunction, and abnormal mitophagy [146]. FIS1 knockdown could reduce mitochondrial autophagy [66]. In contrast to DRP1, there was no significant change in the expression of FIS1 in pMCAO or hyperglycemia model [105]. Paradoxically, one study showed that FIS1 decreased after ischemia/reperfusion, whereas hyperglycemia resulted in a distinct increase of FIS1 expression after 24 h of reperfusion [147]. FIS1 knockout can reduce mitochondrial autophagy and respiration, reducing the number of mitochondria and activating apoptosis [66, 81].

## 5. Mitochondrial Fusion Proteins and Stroke

### 5.1. MFN1/MFN2

The fusion of outer membrane of mitochondria is mediated by MFN1 and MFN2. MFN1 and MFN2 have high homology (81%) and about 60% identity [84, 85]. MFN2 is a GTPase on the mitochondrial outer membrane, which is essential for maintaining intracellular homeostasis [63, 86]. MFN2 was proved to be involved in axonal transport of mitochondria [87], and it also participated in tethering ER to mitochondria [88]. Previous studies have demonstrated that MFN2 was crucial to mitochondrial oxidative phosphorylation: when MFN2 was deleted, mitochondrial oxidative phosphorylation was impaired and ATP production declined [89].

The MFN2 expression has been studied in ischemic stroke in recent years. Cultured primary rat cortical neurons subjected to excitotoxic doses of NMDA or exposed to OGD for 3 h showed reduced expression of MFN2[148]. Likewise, a decrease in the MFN2 expression was also observed in middle cerebral artery occlusion in rats [147]. The expression of MFN2 decreased in C57BL/6 mice subjected to permanent middle cerebral artery occlusion for 12 or 24 h in vivo and in the hypoxia model of cobalt chloride simulating immortalized hippocampal neurons in vitro. However, hypoxia induced apoptosis was improved when MFN2 was restored by using lentivirus [149]. In mouse hippocampal HT22 cells, MFN2 decreased after 10 h of CoCl_2_ induced hypoxia injury, whereas MFN2 increased after 24 h of hypoxia, indicating that CoCl_2_ resulted in a transient decline of mitochondrial fusion [150]. OGD significantly reduced MFN2 in HT22 cells, but the expression of MFN1 did not change [151]. In normal and hyperglycemic ischemic animals, the deletion of mitochondrial uncoupling protein 2(UCP2) significantly reduced the immunoreactivity of OPA1 and MFN2, indicating that the deletion of UCP2 inhibited mitochondrial fusion [100]. As one of the risk factors of ischemic stroke, hyperglycemia can aggravate ischemic brain injury and inhibit mitochondrial fusion. The change of the MFN2 expression in ischemic stroke may provide a research direction for the treatment of ischemic stroke.

When SAH occurs, the expression of Sirtuin3 (SIRT3), a NAD^+^ dependent deacetylase, was downregulated; however, SIRT3 agonist honokiol could increase the levels of MFN1 and MFN2, then maintaining mitochondrial morphology, protecting mitochondrial function, and alleviating brain edema and neurobehavioral defects [152]. Previous research implicated that SIRT3/AMPK is a positive feedback pathway [153]. The activation of AMPK pathway, which plays a key role in regulating cellular energy metabolism including mitochondrial biogenesis, glucose/fatty acid metabolism, and adaptive thermogenesis [154], can elevate the expression of both MFN1 and MFN2 [155]. However, the treatment of an AMPK inhibitor, compound C, can eliminate the protective effect of honokiol on mitochondrial morphology [152]. All of these demonstrated that the activation of SIRT3/AMPK pathway is favor to the protective response of honokiol on mitochondrial fusion in SAH.

The expression of MFN2 in stroke involves a variety of molecular mechanisms. Downregulation of MFN2 leads to mitochondrial dysfunction, ER stress, destruction of calcium homeostasis, and oxidative stress and enhanced the translocation of Bax to mitochondria [156–158], causing delayed neuronal death [159]. The absence of MFN2, which has been demonstrated to stimulate Bax oligomerization, promotes outer mitochondrial membrane remodeling by DRP1 [97]. Changes in MFN2 level facilitates Bax assembly and induces mitochondrial outer membrane permeabilization by affecting the equilibrium of interactions between pro and antiapoptotic different members of the Bcl-2 family [160]. In addition, MFN2 interacts with NLRP3 through its hydrophobic heptad repeat region to promote NLRP3 to recruit IL-1*β* secretion [161]. Knockdown of MFN2 decreased NLRP3-dependent caspase-1 activation and production of IL-1*β* [161]. MFN2 has been shown to have antiapoptotic effect and its expression is reduced under hypoxia [149, 162]. Inhibiting mitochondrial fusion promotes apoptosis. Bak and Bax, members of Bcl-2 protein family, regulate mitochondrial fusion rate by interacting with MFN2 and thus maintain normal mitochondrial morphology[163–166]. The loss of MFN2 contributes to the enhancement of ischemia/reperfusion injury [167]. In addition, MFN2 can influence mitochondrial morphology and participate in mitochondrial autophagy. PINK1-Parkin can ubiquitinate MFN2, and the biological effect can be amplified by phosphorylated MFN2 [168]. Ubiquitinated MFN2 inhibits mitochondrial fusion via increased proteasome degradation, leading to mitochondrial fragmentation and promoting mitochondrial autophagy. PKA inhibition rescued mitochondrial morphology and facilitated optic nerve head astrocyte survival via increasing the oligomerization of MFN1/MFN2 against oxidative stress accompanied by cyclic adenosine monophosphate(cAMP) elevation [169]. There is evidence that MFN2 activation may protect organelles against permeability transition that is closely associated with programmed cell death, interfere with Bax activation, reduce the susceptibility to free radical induced depolarization, and thus play a neuroprotective role in stroke injury [170]. Understanding the molecular mechanism of MFN2 in stroke may contribute to have a deeper insight on pathological process of stroke and provides some therapeutic targets for the treatment of stroke.

### 5.2. OPA1

OPA1 mediates the fusion of mitochondria inner membrane. OPA1 belongs to the dynamin family and has three conserved domains: a GTPase domain, an intermediate domain, and a carboxyl terminal coil domain also known as GTPase effector domain [90]. Different from other dynamins, OPA1 has a variety of isoforms, which must be processed from long membrane anchored form (L-OPA1) to short soluble form (S-OPA1) via protease cleavage to achieve effective fusion of mitochondrial inner membrane [91]. S-OPA1 cannot trigger the fusion of mitochondrial inner membrane alone but rather must cooperate with L-OPA1 [92, 93]. S-OPA1 promotes membrane fusion by oligomerization with L-OPA1, which supports the hydrolysis of GTP and the conformational change of L-OPA1 [91].

There is substantial evidence that the expression of OPA1 decreases after hypoxia injury [150], which is also observed after ischemia/reperfusion injury in vivo [171]. Cerebral ischemia inhibited the expression of OPA1 at 24 h and 72 h and suppressed MFN2 at 6-72 h of recovery in vivo [172]. Moreover, the expression of OPA1 further reduced under hyperglycemia [106]. The depletion or knockdown of OPA1 leads to mitochondrial rupture, cristae structure disorder, and the initiation of apoptosis [173–175]. In addition, the deletion of UCP2 further reduced the level of OPA1, inclining the dynamic balance of mitochondria to fission [100]. The loss of OPA1 function via silencing, gene knock-out, and dominant-negative isoform expression leads to an inhibition of fusion, subsequent the upregulating of mitochondria fission, and increased susceptibility to cell death induction [176]. Importantly, it has been reported that the control of transgenic overexpression of OPA1 can “tighten” the cristae junction, limit the release of cytochrome c, and provide protection against ischemic brain injury after stroke [177]. Together, all studies suggest that OPA1 is a potential target for treatment of ischemic stroke.

Consistently, mitochondrial dynamics tends to mitochondrial fission accompanied by mitochondrial fragmentation, and the expression of OPA1 decreased after SAH [178, 179]. Epigallocatechin-3-gallate (EGCG), a bioactive polyphenol compound with antioxidant activity, can induce normal autophagy flux at the initiation and formation stages via regulating autophagy related 5(ATG5) and beclin-1 to as to eliminate damaged mitochondria timely, and downregulate calcium concentration to regulate the expression of mitochondrial fission and fusion genes to balance mitochondrial dynamics [179]. Therefore, maintaining the balance of mitochondrial fission and fusion cycle and activating autophagy has therapeutic significance for SAH.

**Figure 2:**
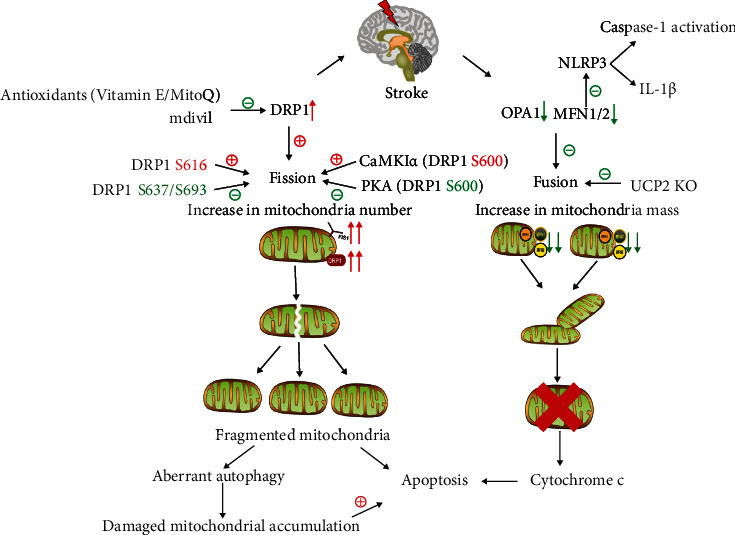
The role of mitochondria dynamin in stroke. Mitochondrial fission proteins DRP1 and FIS1 will increase following stroke, accompanied by the decrease of mitochondrial fusion proteins MFN1/2 and OPA1. The balance between mitochondrial fission and fusion is broken, inclining mitochondrial dynamic to fission. Regulating different phosphorylation sites of DRP1 affects the recruitment of DRP1 in mitochondria, which plays different functional and morphological effects. After stroke, damaged mitochondria accumulate excessively, and mitochondrial fusion is impaired, resulting in mitochondrial autophagy and neuronal apoptosis.

OPA1 plays an important role in stroke. OPA1 can attenuate cerebral edema after ischemic stroke [180]. Similarly, enhancing the expression of OPA1 plays a neuroprotective role in SAH injury [56]. OPA1 has two known functions: maintaining cristae junction and mitochondrial fusion. The induction of neuronal death is mediated by the release of cytochrome *c* from mitochondria, which is controlled by cristae junctions gated by OPA1 oligomer [176]. Blocking the expression of OPA1 by siRNA resulted in the morphological changes of mitochondrial cristae and the production of vesicle structure [181]. OPA1 is also vital for the assembly and stabilization of mitochondrial respiratory chain and the isolation of proapoptotic cytochrome molecules in these tight junctions [177]. Inhibition or mutation of OPA1 results in impaired mitochondrial function, fragmentation, cytochrome c release, cristae remodeling, and apoptosis [172, 174]. Exercise preconditioning can alleviate the brain edema and improve the neurological deficit after ischemic stroke through facilitating the expression of OPA1 and mitochondrial complex II/III/IV closely related to ATP generation [180]. After SAH, MitoQ (a mitochondrial-targeting drug) can promote the attenuation of BBB destruction and the improvement of related neurological deficit via NF-E2-related factor 2 (Nrf2)/prohibitin 2 (PHB2)/OPA1 pathway[178]. Therefore, maintaining the integrity of mitochondrial morphology is an important therapeutic target for stroke (Figure 2).

## 6. Mitochondria Dysfunction, ER Stress, and Ca^2+^ in Stroke

Mitochondria are the repository of calcium, keeping the levels of cytosolic calcium low [182]. ER is a critical organelle that stores and releases Ca^2+^, involved in the maintenance of Ca^2+^ homeostasis [183]. Calcium overload is one of the important pathological mechanisms of cell death caused by stroke [184]. Under severe stress conditions including stroke, excessive accumulation of intracellular Ca^2+^ can lead to the massive release of glutamate, mitochondrial dysfunction, ER stress, neuronal apoptosis, and cell death [185, 186].

After stroke, mitochondrial dysfunction, accompanied by increased ROS production, can cause ER stress, followed by the release of Ca^2+^ from ER into the cytoplasm [187, 188]. The increased free Ca^2+^ in the cytoplasm is transported to the mitochondrial matrix by the Ca^2+^ uniporter. The dysregulation of Ca^2+^ level induces the formation of MPTP, allowing the penetration of toxic mitochondrial matrix molecules into the cytoplasm and eventually causing apoptosis [189]. Disturbance of Ca^2+^ homeostasis can cause neuronal cell damage in stroke [190]; so, maintaining Ca^2+^ homeostasis may be a potential therapeutic target for poststroke neuroprotection. It was demonstrated that transient receptor potential melastatin 2 (TRPM2), a nonselective cation channel with calcium permeability, can be activated under oxidative stress, increasing calcium concentration, which was detrimental in cerebral ischemic stroke[191–194]. The inhibition of endogenous TRPM2 can suppress Ca^2+^ influx and subsequently cell death induced by ischemic stroke in vivo and in vitro[195–198]. Ischemic preconditioning can induce store-operated calcium entry (SOCE) and the release of Ca^2+^, preventing the downregulation of stromal interacting molecule 1(STIM1) and calcium-release-activated-calcium channel protein 1(ORAI1) induced by ischemia-reperfusion injury and restoring the Ca^2+^ homeostasis of ER, thereby playing a neuroprotective role [199].

Ca^2+^ is one of the vital signals of the interaction between ER and mitochondria [200, 201]. Mitochondria and Ca^2+^ mutually regulate each other. Maintaining calcium homeostasis is essential for the balance of mitochondrial dynamics [202].

## 7. Conclusions and Challenges

Mitochondrial dysfunction is the initial event of stroke. Mitochondria are involved in the pathology of stroke by inducing oxidative stress, elevating ROS generation, impairing calcium homeostasis, and controlling cell death. The maintenance of the mitochondria function largely depends on the balance of mitochondrial dynamics. The imbalance of mitochondrial fission and fusion after stroke may increase mitochondrial fragmentation, cause aberrant mitochondrial morphology and disruption of mitochondrial homeostasis, and thus eventually trigger neuronal death. Proper control of mitochondrial fission and fusion is essential for maintaining mitochondrial function and overall cellular health. The clinical treatment of stroke is still a great challenge. Targeting mitochondrial dynamics and regulating the expression of mitochondrial dynamin seem to provide a therapeutic basis for stroke. Therefore, understanding the changes of mitochondrial fission and fusion protein in stroke and the intervention mechanism can provide a new research direction for the prevention and treatment of stroke. However, the discovery of mitochondrial dynamics targeted drugs is still an important challenge for stroke research. The search for new drugs that can improve the clinical prognosis of stroke also needs to be further explored.

## Figures and Tables

**Table 1:** Mitochondrial dynamic-related proteins.

Mitochondria dynamics	Function	Proteins	Location	Modification	Reference
Mitochondria fission	Isolating dysfunctional mitochondria	DRP1	Translocated from the cytoplasmic pool to the outer membrane of mitochondria during fission	S-nitroylation, SUMOylation, ubiquitination, and phosphorylation	[69, 73–79]
		FIS1	Mitochondrial outer membrane	DRP1 recruitment factor, together with MFF, MID49, and MID51	[80–83]
Mitochondria fusion	Avoiding damage accumulation caused by mutations in mitochondrial DNA aging	MFN1/MFN2	Mitochondrial outer membrane	Phosphorylation and ubiquitination	[63, 84–89]
		OPA1	Mitochondrial inner membrane	Protease cleavage: L-OPA1 and S-OPA1	[90–93]

DRP1: dynamin-related protein 1; FIS1: fission protein 1; OPA1: optic atrophy 1; MFN1: mitofusin-1; MFN2: mitofusin-2; MFF: mitochondrial fission factor; MID49: mitochondrial dynamic proteins of 49 kDa; MID51: mitochondrial dynamics proteins of 51 kDa.
